# Research advances in the study of sleep disorders, circadian rhythm disturbances and Alzheimer’s disease

**DOI:** 10.3389/fnagi.2022.944283

**Published:** 2022-08-17

**Authors:** Xiangyang Xiong, Tianpeng Hu, Zhenyu Yin, Yaodan Zhang, Fanglian Chen, Ping Lei

**Affiliations:** ^1^Department of Geriatrics, Tianjin Medical University General Hospital, Tianjin, China; ^2^Tianjin Neurological Institute, Tianjin, China

**Keywords:** sleep disorders, circadian rhythm disturbances, Alzheimer’s disease, amyloid-β, tau

## Abstract

Although there are still no satisfactory answers to the question of why we need to sleep, a better understanding of its function will help to improve societal attitudes toward sleep. Sleep disorders are very common in neurodegenerative diseases and are a key factor in the quality of life of patients and their families. Alzheimer’s disease (AD) is an insidious and irreversible neurodegenerative disease. Along with progressive cognitive impairment, sleep disorders and disturbances in circadian rhythms play a key role in the progression of AD. Sleep and circadian rhythm disturbances are more common in patients with AD than in the general population and can appear early in the course of the disease. Therefore, this review discusses the bidirectional relationships among circadian rhythm disturbances, sleep disorders, and AD. In addition, pharmacological and non-pharmacological treatment options for patients with AD and sleep disorders are outlined.

## Sleep disorders, circadian rhythm disturbances and Alzheimer’s disease

**FIGURE 1:**
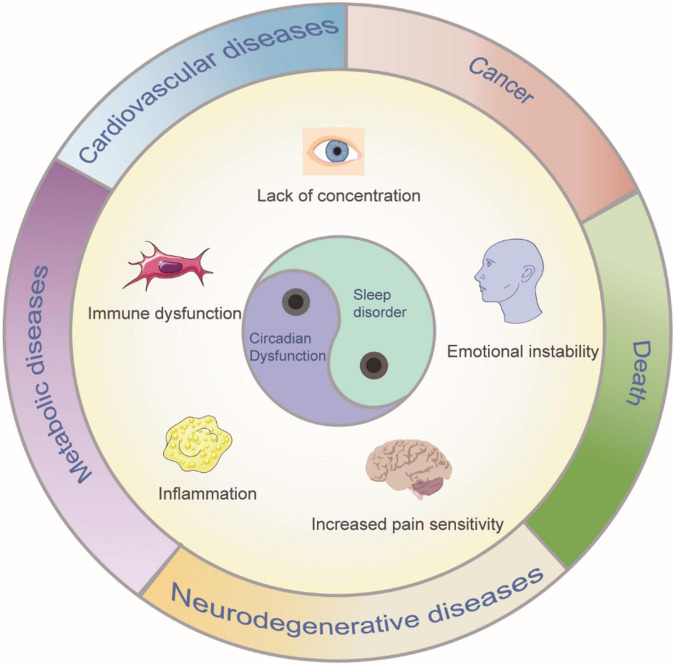
Sleep disorder and circadian dysfunction may lead to clinical symptoms and diseases. Such consequences can include poor concentration, emotional instability, increased sensitivity to pain, metabolic, and cardiovascular diseases, immune dysfunction and even, in extreme cases, death.

Sleep is induced by neurons, therefore, it is believed that death due to sleep deprivation is the result of impaired brain function. In a very early study, puppies that were completely deprived of sleep died within days, with the most serious lesions occurring in the brain. Through a histological study of the dead puppies, degenerative changes in spinal ganglion neurons, cerebellar Purkinje cells and frontal cortex neurons were observed (Bentivoglio and Grassi-Zucconi, 1997). In a recent study, the movement of chromosomes (chromosome dynamics) in the neurons of zebrafish was photographed at regular intervals; sleep was found to triple the chromosome dynamics. In general, sleep increases chromosome dynamics, which changes the structure of chromosomes and reduces DNA damage, thus reducing death (Zada et al., 2019). Additionally, a human clinical study found a decrease in temporal lobe nerve activity after sleep deprivation (Nir et al., 2017). Sleep is an active process: specific brain structures are activated during specific stages of sleep and deactivated during other stages. Normal sleep stages includes non-rapid eye movement (non-REM) sleep and rapid eye movement (REM) sleep, which have different EEG characteristics (Tamaki et al., 2020). A sleep cycle lasts approximately every 90 min, and these cycles occur four to five times a night (Tamaki et al., 2020). Sleep is typically necessary for brain activity. By promoting the clearance of brain metabolites, sleep plays a key role in maintaining the dynamic balance of metabolism (Xie et al., 2013). Sleep can also save energy and help animals escape the notice of predators. Furthermore, sleep is important in consolidating memory (Girardeau and Lopes-Dos-Santos, 2021). The average person needs approximately 8 h of sleep per night, but many otherwise healthy people deprive themselves of adequate sleep, resulting in fatigue, poor decision-making and a higher risk of accidents (Bandyopadhyay and Sigua, 2019). Various studies have shown that chronic sleep deprivation leads to poor concentration, emotional instability (Yoo et al., 2007), increased sensitivity to pain (Alexandre et al., 2017), metabolic and cardiovascular diseases (Broussard et al., 2012; Huang et al., 2020), immune dysfunction and even, in extreme cases, death. A long-term mismatch between circadian rhythms and lifestyle may be associated with a high risk of several diseases, such as neurodegenerative diseases, inflammation, cancer, and metabolic disorders (Hirota and Kay, 2015; Figure 1). These negative effects of sleep deprivation elucidate the significance of sleep. A balanced and adequate sleep cycle is one of the main factors determining human quality of life. Many factors contribute to sleep deprivation, including environmental, psychological, and physical factors. Changes in the environment affect many aspects of our daily lives, and sleep disorders may occur due to increased ambient noise and fluctuations in light and temperature (LeGates et al., 2012; Kroller-Schon et al., 2018). Mental health also plays a crucial role in maintaining regular sleep patterns, and mental illnesses, such as stress, anxiety, and depression, interfere with regular sleep cycles, which may translate into insomnia (O’Loughlin et al., 2021). Additionally, psychiatric disorders such as schizophrenia or neurodegenerative diseases such as Alzheimer’s are often associated with sleep deprivation, such as shortened rapid eye movement sleep cycles and reduced sleep spindle activity (Fernandez and Luthi, 2020). Modern lifestyle changes such as unbalanced work hours, excessive caffeine intake, and increased screen time potentially affect the quality and quantity of sleep. However, the dynamic balance of normal biological circadian rhythms is necessary to achieve better cognitive and task performance. For example, night shift workers experience varying degrees of cognitive impairment. Shift work can forcibly disrupt the normal sleep-wake cycle, resulting in insufficient sleep and excessive fatigue, which, in turn, affects long-term health and safety (Kecklund and Axelsson, 2016). Substance abuse and alcohol intake have also been implicated as the causes of an increasing number of sleep disorders (Guo et al., 2016; Hasler and McClung, 2021). For instance, alcohol consumption inhibits REM sleep, which in turn impairs the performance of routine tasks (Chan et al., 2015). Additionally, sleep deprivation can affect the balance of important neurotransmitters in the brain; one important example is dopamine (DA), the disruption of which has been shown to increase susceptibility to substance use (Hasler and McClung, 2021). The negative effects of sleep deprivation described above encompass only a small portion of these effects, but can help convey the importance of sleep.

Alzheimer’s disease (AD) was first identified in 1907 when the German physician Alois Alzheimer reported a case in a 56-year-old woman, subsequently, the disease was named after him (Alzheimer et al., 1995). Currently, approximately 44 million people worldwide have AD or related dementias, and that number is expected to reach approximately 135 million by 2050 (Hossain et al., 2019). Typical features of AD are the gradual deterioration of memory, language and cognitive function. Sleep and circadian rhythm disturbances are very common in patients with AD, and up to 45% of patients experience sleep problems. The clinical presentation of AD is characterized by memory loss and cognitive dysfunction. With the increase of age, changes in the brain as well as in other cells of the body are evident. Examples include changes in the genome and epigenome (Schumacher et al., 2021), loss of protein balance (Hetz, 2021), a decline in cellular and subcellular function (Bulati et al., 2017), and deregulation of signaling systems (Martinez-Jimenez et al., 2017). Most people gradually experience difficulties such as losing the ability to think and/or remember certain things and begin to notice these changes. However, difficulty remembering new information is the most common early feature of AD because in the initial stages, changes occur in areas of the brain involved in learning. In the later stages of AD, changes in the brain lead to increasingly severe symptoms, including disorientation, behavioral and mood changes, increasing uncertainty about events, places and times, unfounded doubts about family, friends and professional caregivers, severe memory loss, and difficulties in everyday tasks such as swallowing, speaking and walking. The prevalence of AD has progressively increased since the 1950s, this disease has a duration of 1–20 years or more and shortens life expectancy (Selkoe, 2013).

**FIGURE 2:**
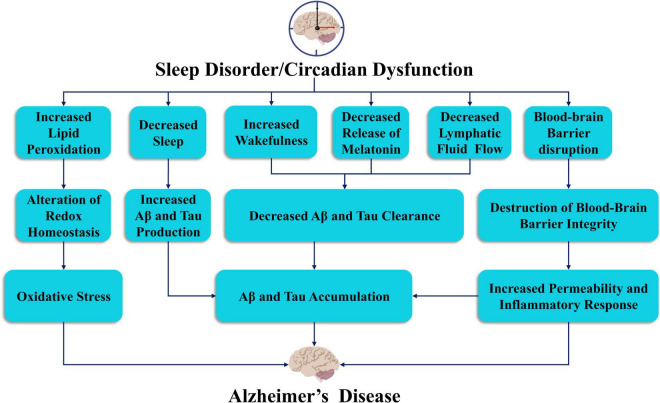
The link between sleep deprivation and circadian dysfunction in the pathogenesis of Alzheimer’s disease. Sleep and circadian rhythm disorders may lead to oxidative stress, accumulation of Aβ and tau proteins, decreased release of melatonin, reduced lymphatic flow, and damage to the blood-brain barrier, which may lead to the development of AD.

Growing evidence for the role of sleep disorder in the pathophysiology of AD has resulted in the proposal of a bidirectional relationship (Ju et al., 2014; Guarnieri and Sorbi, 2015; Villa et al., 2015). The identification of sleep disorders and circadian rhythm disturbances can serve as initial markers for AD diagnosis (Saeed and Abbott, 2017). The pathogenesis of AD is still unclear, though the most influential factors appear to be extracellular plaque deposits of β-amyloid (Aβ) and tau-containing intracellular neurofibrillary tangles (NFTs) (Knopman et al., 2021; Figure 2). Aβ is derived from amyloid precursor protein (APP), which is cleaved by β-secretase and γ-secretase, and NFTs, which are aggregates of hyperphosphorylated tau proteins. However, autopsy of these classic pathological features only explain the manifestation of dementia to a limited extent (Matthews et al., 2009). The risk of AD attributable to heredity is approximately 70%. Earlier studies have identified genetic causes of AD, which include dominant mutations in the genes encoding APP, Presennilin-1 and Presennilin-2 (Goate et al., 1991; Schellenberg et al., 1992; Levy-Lahad et al., 1995). These genes are critical to our understanding of the pathogenesis of AD. Sortilin-related receptor 1 (SORL1) is also thought to be an important genetic cause of late-onset AD (Rogaeva et al., 2007). In addition, several genes that are potential risk factors for AD have been identified. The most strongly associated risk genes were apolipoprotein E (ApoE) (Corder et al., 1993; Uddin et al., 2019), glycogen synthase kinase 3β (GSK3β) (Hernandez et al., 2009), dual specificity tyrosine-phosphorylation-regulated kinase 1A (DYRK1A) (Kimura et al., 2007), translocase of outer mitochondrial membrane 40 (TOMM40), clusterin (CLU) (Thambisetty et al., 2010) and phosphatidylinositol-binding clathrin assembly (PICALM). These genes may play an important role in identifying pathways and potential drug targets associated with AD. However, recent studies have shown that certain individuals do not need at least 7 h of sleep per night due to their genetics. This group of short sleepers need only 4 or 5 h of sleep each night to be energized and refreshed and thus exhibit a reduction in required sleep throughout their lifetime. More importantly, this reduction in sleep does not impact their cognitive function or induce AD. The short sleep ability is associated with mutations in the hereditary natural short sleep gene, in which the development of tau pathology is slowed according to findings in mice carrying mutations in the DEC2-P384R and Npsr1-Y206H short sleep genes (Dong et al., 2022).

In this review, we explore the evidence linking sleep disorders, circadian rhythm disorders and AD, focusing on the complexity of the main mechanisms underlying the relationship between sleep disorders and AD. In addition, this review highlights potential therapeutic strategies for reducing sleep disorders in patients with AD. We review evidence that sleep affects the pathogenesis of AD and summarize three key research areas: (1) the potential risks of sleep disorders for Alzheimer’s disease, (2) the mechanism by which sleep disorders lead to Alzheimer’s disease, and (3) the treatment of sleep disorders in patients with AD. On the basis of these findings, we recommend highly individualized early interventions. We aim to provide policy makers, researchers and clinicians with necessary background information to fully understand this review’s clinical relevance, the potential pathophysiology of AD, and potential methods of early diagnosis and prevention to establish better treatment in terms of personalized care and protection of elderly individuals.

## Sleep disorders are a potential risk factor for Alzheimer’s disease

Various studies have shown that sleep is a natural and important biological activity and that sleep disorders can lead to neurodegeneration that is not limited to progressive disease but occurs before cognitive impairment, and thus, they may be considered a marker of neurodegeneration (Lim et al., 2013; Hahn et al., 2014).

In the brain, many areas, neurotransmitters and trigger switches strictly control the sleep-wake cycle. Sleep regulatory areas are distributed throughout the brain, including the brainstem, midbrain, thalamus, hypothalamus, and basal forebrain (Saper et al., 2005). These regions regulate arousal, promote and inhibit NREM and REM sleep, and regulate circadian rhythms. A study reported that slowing circadian rhythms in healthy, community-dwelling older women increases the odds of developing mild cognitive impairment (MCI) and dementia (Tranah et al., 2011). This conclusion was confirmed in a recent study (Posner et al., 2021). In fact, weaker circadian rhythms are also associated with lower cognitive function, especially in older women without dementia (Walsh et al., 2014). Additionally, sleep disorders are the leading cause of AD, and most people with AD exhibit some type of sleep disorder (Cui and Li, 2013; Cui et al., 2015). A previous study clearly emphasized the importance of sleep, as sleep deprivation in rats led to death within 2–3 weeks (Rechtschaffen, 1998).

In recent years, the interaction between sleep disorders and AD has attracted increasing attention. Accumulating evidence indicates that many diseases, including AD, multiple sclerosis, and Parkinson’s disease (PD), are directly associated with sleep disorders (Barun, 2013; Mendelsohn and Larrick, 2013; Ondo, 2014). Moreover, sleep disorders are not only an important clinical feature of AD but also an important risk factor. Targeted sleep regulation has become an important strategy and therapeutic target for AD prevention and treatment.

## Study on the mechanism of sleep disorders leading to Alzheimer’s disease

### Sleep disorders affect Aβ aggregation, leading to the onset of Alzheimer’s disease

Sleep-wake cycles and circadian rhythms play a critical role in controlling Aβ levels (Figure 2). Both mouse and human sleep disorders increase Aβ levels in the brain and potentially increase plaque deposition (Kang et al., 2009; Ooms et al., 2014; Ju et al., 2017). Moreover, Aβ levels exhibit a significant circadian rhythm in both mouse and human models, and Aβ in the interstitial fluid (ISF) of the brain is positively correlated with wakefulness, negatively correlated with sleep duration, and significantly negatively correlated with the amount of NREM sleep (Holth et al., 2019). In addition, levels of Aβ in human cerebrospinal fluid (CSF) samples were also found to be highest during wakefulness and lowest during sleep, with a difference of up to 30% between the two levels (Lucey et al., 2018). Previously, the central nervous system was widely believed to lack lymphatic circulation, but this view has been updated upon the discovery of a network of meningeal lymphatic vessels in rodents, non-human primates, and human dura mater (Aspelund et al., 2015; Louveau et al., 2015; Absinta et al., 2017). The lymphatic system of mice has also been shown to participate in the removal of solutes, including Aβ, from the ISF of mice (Iliff et al., 2012). Apparently, sleep, whether normal or induced by anesthetics, increases ISF space in the brain by 60%, which is essential for the clearance of toxic metabolites such as Aβ (Xie et al., 2013). If animal models experience persistent insomnia, they die within days or weeks (Kress et al., 2014). This finding was also observed in patients with fatal familial insomnia (Kress et al., 2014). In addition, sleep deprivation increases the accumulation of Aβ in transgenic mice, conversely, proper sleep reduces Aβ levels and enhances clearance (Xie et al., 2013). These findings suggest that sleep disorders increase the risk of developing AD by increasing the production of Aβ. The relationship between AD and Aβ has been recognized in preclinical and clinical studies, but Aβ accumulation can also be found in some cognitively healthy people (Davis and Chisholm, 1999; Price et al., 2009). Conversely, neurodegeneration can also occur without plaque accumulation (Chetelat, 2013). Indeed, Aβ plaques may not be the only cause of AD, and there may be no obvious causal relationship between them (Murphy and LeVine, 2010; Figure 2). Clinical drug development for Aβ as a therapeutic target has failed, which urges us to further study the mechanism of AD and whether Aβ is a final metabolite rather than the initiator of the pathophysiological process.

### Sleep disorders affect tau levels, leading to Alzheimer’s disease onset

Although substantial evidence suggests that Aβ aggregation initiates the pathogenesis of AD, such as by driving tau protein aggregation and diffusion, tau aggregation appears to promote neurodegenerative lesions (Simic et al., 2016; Figure 2). The researchers measured tau protein levels in the ISF and CSF to examine whether tau levels were affected by the sleep-wake cycle and sleep deprivation. The results showed that the tau level in the ISF in mice increased by approximately 90% in the normal waking state and by 100% in the sleep deprivation state. In humans, tau levels in the CSF also increased by more than 50% during sleep deprivation. Thus, the sleep-wake cycle regulates tau levels in the ISF, and sleep deprivation increases tau levels in the ISF and CSF as well as pathological diffusion (Holth et al., 2019). In addition, dysfunctional tau protein metabolism has been observed in circadian rhythm disturbances and sleep disorders (Barthelemy et al., 2020). Many brain regions involved in sleep regulation, such as the parabrachial nucleus, locus coeruleus, midbrain periaqueductal gray, dorsal raphe nucleus, lateral hypothalamic nucleus, tuberomammillary nucleus, and basal forebrain, are characterized by different tau-related pathologies. Unlike Aβ levels, total and phosphorylated tau levels in the CSF predict cognitive decline in preclinical and clinical AD (Fagan et al., 2007; Mattsson et al., 2009). Furthermore, tau pathology has recently been shown to be the earliest observable AD-like change in the human brain, with abnormal tau phosphorylation and aggregation in the locus coeruleus beginning in early adulthood (Braak et al., 2011) and extending to other connected regions even before amyloid is detected (Stratmann et al., 2016). Recent studies have also found that the norepinephrine metabolite DOPEGAL covalently modifies the K353 site of the tau protein in the locus coeruleus, promoting tau aggregation and the spread of lesions (Kang et al., 2022). Despite these observations, little is known about the impact of tau pathology on sleep in preclinical or clinical AD. These areas highlight the important role of tau in AD sleep disorders (Figure 2).

### Other factors potentially leading from sleep disorders to Alzheimer’s disease

One study induced chronic REM sleep disorders in mice by chronic sleep fragmentation (SF), which enhanced neural activity in the medial habenula (MHB), a brain region increasingly implicated in negative effects. Cholinergic neurons (CHNs) in the MHB exhibited higher spontaneous firing rates and enhanced regularity of brain slice firing after 5 days of chronic SF. When glutamate, GABA, acetylcholine, and histamine receptors were inhibited, the changes in SF-induced firing were unchanged, indicating that this autonomic mechanism was independent of synaptic transmission. In addition, the SF-induced hyperactivity of CHNs in the medial habenular nucleus was not due to the enhancement of the excitability of the lamina propria but was accompanied by depolarization of the resting membrane potential. SF impaired the function of TASK-3 in CHNs in the medial habenular nucleus, which may have led to the depolarization of the resting membrane potential and spontaneous firing. These results not only proved that REM sleep disorder leads to hyperactivity of CHNs in the MHB but also identified the key molecular substrates affected by REM sleep disorder. Chronic REM SF induces CHN hyperactivity in the MHB by regulating the K_2P_ channel TASK-3. Sleep disorders may thus affect mood regulation through this molecular mechanism, which may induce neurodegenerative diseases (Ge et al., 2021).

With increasing age, the interruption of the sleep-wake cycle is accompanied by increasing oxidative stress, which indicates that the accumulation of age-related oxidative damage may lead to the interruption of the sleep-wake cycle (Koh et al., 2006; Figure 2). The latest research has also shown that mortality caused by severe sleep deprivation may be due to oxidative stress, particularly in the intestinal tract. Sleep deprivation can lead to death through the accumulation of reactive oxygen species (ROS) in the intestinal tract, but this accumulation can be reversed by oral antioxidant compounds or targeted intestinal antioxidant enzymes (Vaccaro et al., 2020).

Melatonin released from the pineal gland can regulate circadian rhythms and prevent additional Aβ accumulation (Figure 2). Previous studies have shown that melatonin levels in the CSF in patients with AD are decreased, even during preclinical AD and disease progression. Recent studies have shown that melatonin directly binds to and inhibits the function of death-associated protein kinase 1 (DAPK1) in AD, thereby reducing the accumulation and phosphorylation of tau protein and promoting synaptic growth and microtubule assembly (Chen et al., 2020). This finding indicates that the loss of melatonin may enhance the development of AD.

The blood–brain barrier (BBB) is a large regulatory and exchange interface between the brain and the peripheral circulation. Sleep deprivation impairs the function of the BBB. Chronic sleep deprivation not only reduced the expression of inducible nitric oxide synthase, endothelin-1, and glucose transporter in BBB microvascular endothelial cells but also reduced brain uptake of 2-deoxy-D-arabino-hexose. Increased inflammation associated with COX-2 altered vascular reactivity, decreased glucose transporters and impaired BBB permeability, which led to the occurrence of AD (He et al., 2014; Figure 2).

## Treatment of sleep disorders in patients with Alzheimer’s disease

**FIGURE 3:**
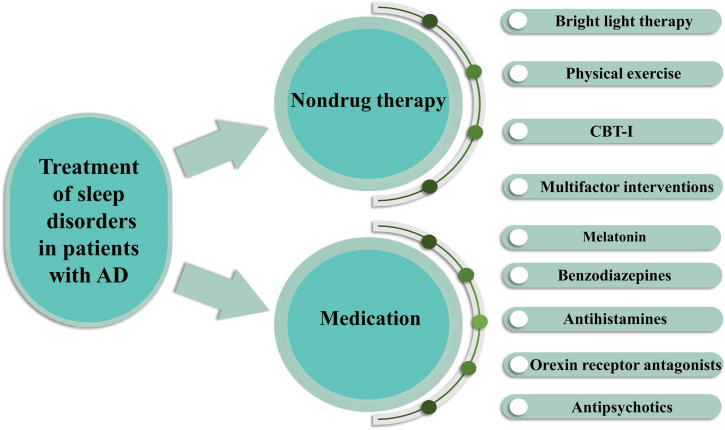
Treatment of sleep disorders in AD patients by pharmacological and non-pharmacological means. Non-drug therapy includes bright light therapy, physical exercise, cognitive behavioral therapy for insomnia (CBT-I) and multifactor interventions. Drug treatments include melatonin, benzodiazepines, antihistamines, orexin receptor antagonists, antipsychotics.

The treatment of sleep disorders in patients with AD aims to improve the quality of life of patients and caregivers. Because of the impact of sleep disorders on AD, systematic clinical evaluation is essential for selecting the most appropriate treatment for each patient (Figure 3).

### Non-drug therapy

#### Bright light therapy

Non-drug therapy is typically considered a first-line treatment for patients with AD, although there is limited scientific evidence as to its effectiveness. However, in a structured literature review, BLT was shown to be an effective treatment (Brown et al., 2013; Figure 3). Currently, the mechanism by which bright light therapy (BLT) treats sleep disorders in patients with AD is believed to be through retinal neurons; these retinal neurons contain a photosensitive pigment that can transmit light signals to the suprachiasmatic nucleus (SCN), which is the epicenter of circadian rhythms and regulates the secretion of melatonin (Wen et al., 2020). Melatonin synchronizes endogenous circadian rhythms, and its synthesis and secretion are affected by the photoperiod (Shirani and St, 2009). After stimulation from a light source, photosensory ganglion cells in the retina alter the neurotransmitter (e.g., 5-hydroxytryptamine, dopamine, and norepinephrine) and neuroendocrine levels (e.g., sex hormones, thyroid hormones, and melatonin) as well as the neuroimmune system (e.g., IL, TNF-α, and IFN) through visual and non-visual pathways; these changes further influence the mental state and behavior of patients with AD (Maynard et al., 2017). Among these pathways, the visual pathway involves photoreceptors that receive light stimuli, which act on intrinsically photosensitive retinal ganglion cells (ipRGCs); these signals are transmitted to the hypothalamic suprachiasmatic nucleus through the retinal hypothalamic tract, thus regulating a variety of light-related physiological processes, such as stimulating the expression of circadian-related genes and proteins, affecting spontaneous action potential, and regulating pineal secretion of melatonin (Shirani and St, 2009; Sonoda et al., 2020). Therefore, BLT influences biological circadian rhythms, mood and sleep in ways that improve the sleep quality of patients with AD. The wavelength, dose and optimal timing of light therapy are closely related to its therapeutic effect (Shirani and St, 2009). In a 24-week cluster randomized trial, the effect of BLT on the emotion of dementia patients was greater than that of the control group after 16 weeks, suggesting that BLT may be beneficial to dementia patients (Kolberg et al., 2021). However, a new systematic review of the application of BLT in elderly patients with cognitive impairment shows that while BLT improves the management of sleep, behavioral and emotional disorders in patients with cognitive impairment, its impact on cognition, quality of life and functional status is limited (Cibeira et al., 2020). The American Academy of Sleep Medicine published practical parameters for the use of bright light to treat sleep and circadian rhythm disturbances 10–20 years ago (Chesson et al., 1999; Morgenthaler et al., 2007), however, there is no recognized gold standard for bright light exposure, including when to implement it, the duration, the safest and most effective wavelengths of light, or the best mode of exposure. There is increasing interest in the efficacy of BLT alone or in combination with other therapies for treating sleep disorders in patients with AD. However, the systematic review reported great deal of heterogeneity. Therefore, future studies should aim to determine the optimal intensity, frequency and duration of BLT interventions and assess their effectiveness.

#### Physical exercise

Regular exercise is an important measure to prevent the development of circadian rhythm disturbances and stimulates resynchronization of circadian rhythms (Figure 3). The potential mechanism underlying the relationship between exercise and sleep is a complex physiological process involving multiple systems. However, acute and chronic exercise exert different effects on sleep according to the mode, intensity, duration, and dosage of exercise as well as patient age and sex (Driver and Taylor, 2000). Exercise promotes sleep by regulating the central nervous system, endocrine system, metabolism, and body temperature during sleep (Uchida et al., 2012). Exercise can lead to a significant increase in the duration of stages 3 and 4 of slow wave sleep (SWS) (Hori et al., 2001). Habitual exercise enhances vagus nerve regulation, leading to bradycardia, which reflects enhanced parasympathetic nerve control (Sandercock et al., 2005) and may improve sleep and mood through monoamine activation, hypothalamus-pituitary-adrenal axis activation or expression of brain-derived neurotrophic factor (BDNF) (Brosse et al., 2002). Exercise affects thermoregulation (McGinty and Szymusiak, 1990) and body recovery (Adam and Oswald, 1983), according to the energy conservation hypothesis (Berger and Phillips, 1988), promoting sleep. Timely exposure to circadian phase-shifting stimulation can not only normalize circadian rhythms but also improve sleep (Campbell et al., 1993). Therefore, exercise in well-lit conditions may improve the sleep of shift workers, air travelers, patients with atypical depression and older people with abnormal circadian rhythms (Driver and Taylor, 2000). The latest research shows that skeletal muscle, an endocrine organ, can release various muscle cytokines during exercise and exert an anti-inflammatory effect (Hoffmann and Weigert, 2017). Aerobic exercise can inhibit neuroinflammation and activation of microglia (Mee-Inta et al., 2019). This form of exercise can also prevent inflammatory reactions and learning and memory impairments caused by acute sleep deprivation and reverse the decline in cognitive function caused by sleep deprivation (Kojima et al., 2020). A previous randomized controlled study showed that elderly people with moderate sleep problems improved their sleep quality with regular moderate-intensity exercise (King et al., 1997). Studies have also shown that participating in non-strenuous daytime activities can have a beneficial effect on sleep in individuals with dementia. In one study, compared with the control group, dementia patients with severe sleep disorders randomly received 15–30 min of personalized social activities every day for 21 consecutive days, which significantly reduced their daytime sleepiness, sleep latency and nocturnal awakenings (Richards et al., 2005). Another study showed that 30 min of collective activity twice a week for 12 weeks improved sleep and increased the total sleep duration compared with the control group by significantly reducing the number of nighttime arousals after falling asleep. In addition, moderate physical activity has been suggested to improve the sleep of patients with AD (Joranson et al., 2021). Although there is plenty of evidence that increases in physical activity can significantly improve sleep and cognitive abilities in patients with AD, health care professionals rarely systematically advise physical activity to prevent or treat diseases that affect the brain. Physical activity decreases with age, especially among women, low-income people, and in some ethnic groups (August and Sorkin, 2011). Notably, these people are disproportionately affected by AD, so clinicians should discuss the benefits of physical activity for maintaining brain health.

#### Cognitive behavioral therapy for insomnia

The American College of Physicians (ACP) recommends Cognitive behavioral therapy for insomnia (CBT-I) as the initial treatment for chronic insomnia (Qaseem et al., 2016). The ACP also recommends that clinicians and patients use a standardized decision-making approach, including discussion of benefits, hazards, and costs, to decide whether to use drugs when CBT-I alone does not work (Qaseem et al., 2016). CBT-I is a multimodal therapy that combines several behavioral and cognitive interventions. Specifically, this therapy includes education, stimulus control instructions, bed rest time limits and relaxation training. CBT-I produces reliable, lasting benefits in 70–80% of patients and may reduce the use of sedatives (Trauer et al., 2015). This therapy can be provided by trained therapists or through self-directed, fully automated online programs. Overall, CBT-I has moderate to significant effects on sleep-related outcomes, including time to fall asleep, continuity of sleep, feeling rested, and sleep duration (Trauer et al., 2015). Brief behavioral treatment for insomnia (BBTI) is an evidence-based, easy-to-manage method derived from CBT-I and can also be used in a variety of treatment environments (Buysse et al., 2011). BBTI includes four behavioral interventions to improve sleep consolidation by increasing the “driving force” of sleep, improving sleep regularity, reducing arousal, and increasing the link between being in bed and sleeping as follows: (1) reducing bedtime to match actual sleep onset, (2) getting up at the same time every day, regardless of sleep duration, (3) not going to bed unless sleepy, and (4) not lying in bed unless attempting to sleep (Buysse et al., 2017). The patient’s progress is monitored through daily sleep diaries and weekly phone calls or electronic communications. As sleep becomes more consolidated, patients can gradually increase their bed rest time to achieve the best balance between sleep continuity and sleep duration.

#### Multifactor interventions

Because of the risk of drug side effects, behavioral strategies are often recommended as first-line treatments for patients with dementia and sleep disorders (Figure 3). Standard recommendations include setting a regular bedtime and wake-up time, limiting daytime naps and time spent resting in bed, establishing a regular mealtime, avoiding alcohol, nicotine and caffeine, and emptying the bladder before going to bed. Additionally, the sleep environment should be maintained at an appropriate temperature (not be too hot or too cold) and light and noise should be minimized (McCurry and Ancoli-Israel, 2003). In a randomized controlled trial of a nocturnal insomnia treatment for patients with AD, a combination of sleep health education, moderate physical activity and increased light significantly improved the quality of sleep compared with that of the control group, and this therapeutic effect was maintained until the 6-month follow-up (McCurry et al., 2005). Diet and exercise affect cognitive decline and the risk of dementia through endogenous factors triggered by food metabolism and exercise (e.g., changes in apoptosis and decreased integrity of hippocampal progenitor cells), according to a new study. These endogenous factors travel to the brain through the bloodstream and may interact with the hippocampus to cause cognitive decline and dementia (Du Preez et al., 2022). Therefore, the combination of exercise and diet is a feasible and promising method to reduce or prevent sleep disorders in AD patients.

#### Medication

##### Melatonin

Melatonin, a neurohormone secreted by the pineal gland, has received considerable research interest as an alternative drug for the treatment of sleep dysfunction in neurodegenerative diseases (Figure 3). Initially, using neuroblastoma cell lines exposed to Aβ, researchers found that melatonin had antioxidant and anti-apoptotic effects in AD (Pappolla et al., 1997). Further research found that melatonin can inhibit Aβ (Pappolla et al., 1998). These early studies reflect the increasing interest in melatonin as a potential modification therapy to improve the symptoms of patients with AD and sleep disorders. Subsequent studies have shown that melatonin improves behavioral defects in AD-model mice through antioxidant mechanisms (Raghavendra and Kulkarni, 2001) and that exogenous melatonin has anti-tau activity (Liu and Wang, 2002). Moreover, in AD-model animals, melatonin reduced Aβ levels (Matsubara et al., 2003), reduced neuroinflammation (Gunasingh et al., 2008), and reduced damage to hippocampal neuroplasticity (Stefanova et al., 2015). A recent study showed that melatonin plays an important role in regulating circadian rhythm disturbances by controlling clock genes, it also attenuates Aβ accumulation and tau hyperphosphorylation by regulating the GSK3 and CDK5 signaling pathways (Hossain et al., 2019). Similarly, a previous randomized, double-blind, placebo-controlled, crossover study showed that short-term use of melatonin improved sleep, mood and cognition in elderly individuals with mild cognitive impairment (Jean-Louis et al., 1998). However, in a randomized clinical trial, exogenous melatonin had no effect on objective sleep outcomes in patients with AD, though it did have a positive effect on sleep therapy (Zhang et al., 2016). Other studies have failed to find any benefits of melatonin for sleep quality in patients with AD (Serfaty et al., 2002; Gehrman et al., 2009). These different results may be explained by the limited duration of the trial and the inclusion of different stages of dementia and AD patients. Therefore, the relationship between melatonin and AD-related sleep disorders needs to be further evaluated in future studies.

##### Benzodiazepines

Benzodiazepines are derivatives of 1,4-benzodiazepine and act mainly on the reticular formation of the brainstem and on the limbic system. These drugs reduce sleep latency by activating γ-aminobutyric acid (GABA) receptors, the main inhibitory neurotransmitter receptors in the brain, thus improving sleep quality. Benzodiazepines have become the most widely used sedative and hypnotic drugs in clinical practice. Several early studies have shown that benzodiazepines reduce REM sleep and/or slow wave sleep, with reliable improvements demonstrated in patients with chronic sleep disorders (Nowell et al., 1997), later studies showed that these two sleep stages are essential for memory consolidation (Rasch and Born, 2013; Todorova and Zugaro, 2019) and the clearance of neurotoxic waste (Rasmussen et al., 2018; Figure 3). Conversely, one study found that taking benzodiazepines increased the risk of AD, especially with long-term use (Billioti et al., 2014): there was a 43–51% increase in the risk of developing AD in patients who took benzodiazepines. Further analysis indicated that long-term use of benzodiazepines was associated with a greater risk of developing AD than short-term use, supporting a causal relationship (Yaffe and Boustani, 2014). However, the results from a large cohort study suggest that increased benzodiazepine use is not associated with faster cognitive decline. Those with the lowest exposure to benzodiazepines had a slightly higher risk of developing dementia, but those with the highest level of benzodiazepine exposure did not have a higher risk of developing dementia (Gray et al., 2016). These results do not support a causal relationship between benzodiazepines and dementia. However, given the numerous adverse effects of benzodiazepines, the use of such drugs in the neurodegenerative disease population should be approached with caution.

##### Treatment of sleep disorders in patients with Alzheimer’s disease with other drugs

Antihistamines, especially diphenhydramine, are over-the-counter drugs that are widely prescribed to the elderly. These drugs have significant side effects, including sedation, cognitive impairment, increased daytime drowsiness, and anticholinergic reactions. Because of the risk of side effects, the use of antihistamines should not be the first choice of treatment for elderly individuals. Some studies have found that antidepressants have a significant effect on sleep disorders in patients with AD and they may delay the decline in cognitive function. Orexin receptor antagonists can also improve the total sleep duration of patients with mild to moderate AD-related insomnia (Herring et al., 2020). Antipsychotics are another class of drugs used to treat sleep disorders in neurodegenerative diseases. Studies have shown that the low-dose atypical antipsychotic risperidone improves the 5-year prognosis of AD patients with sleep disorders (Yin et al., 2015; Figure 3).

Because of the complexity of sleep disorders in patients with AD, it is impossible to treat them with only one drug or a combination of drugs. The key strategy is to help patients maintain high quality sleep, delay the decline in cognitive, and behavioral performance, and alleviate specific concerns. Therefore, it is highly important for researchers to establish new treatments for the special genetic, molecular and cellular mechanisms of the disease.

## Summary and prospects

Sleep disorders and circadian rhythm disturbances are very common in patients with AD, the etiology is complex, involving many factors, and these disorders appear early in the course of the disease. This series of problems seriously affects the quality of life of patients, family members and caregivers. To accurately diagnose neurological diseases and develop care plans to improve sleep quality and reduce the burden on caregivers, it is important to provide a comprehensive review of current drugs and the possible causes of sleep changes in patients. Through the combined use of drug and non-drug treatments, early interventions can delay the course of sleep disorders in patients with AD.

Although substantial progress has been made in understanding the basic mechanisms that control the biological clock and the neural circuits involved in sleep, little is known about how these systems are affected in the brain in neurodegenerative diseases, which can differ based on sex, APOE4 status, depression and drug use. A more detailed understanding is needed of the mechanisms by which specific neurodegenerative diseases and pathogenic proteins affect the circadian rhythm and sleep system, as well as the interaction between sleep and the circadian rhythm system in AD. Sleep disorders may appear as an early marker of disease pathology that may exist in the brain years to decades before clinical symptoms appear. The extent to which sleep disorders are a risk factor for the occurrence or progression of neurodegenerative disease is unclear. Other prospective studies using biomarkers such as cerebrospinal fluid and PET Aβ concentrations are needed to help document biological changes associated with sleep disorders. In addition, sleep disorders in the elderly need to be assessed in clinical trials to determine sleep indicators and evaluate cognitive results to indicate whether improved sleep can help prevent or delay the occurrence or progression of dementia. The role of the lymphatic system in neurodegeneration should be another priority. Although the comprehensive study of the circadian rhythm system, sleep and neurodegeneration in the lymphatic system is still in its infancy, there is considerable potential to use these powerful systems to control many key aspects of brain function to prevent neurodegeneration.

## Author contributions

XX, PL, and FC conceived, designed, and drafted the manuscript. XX wrote the original draft preparation. TH, ZY, and YZ contributed to the review and edit of the manuscript. All authors read and approved the final manuscript.
